# Tuning the surface plasmon resonance in gold nanocrystals with single layer carbon nitride

**DOI:** 10.1039/c8ra09454c

**Published:** 2019-01-02

**Authors:** O. Stroyuk, A. Raevskaya, G. Grodzyuk, N. Andriushina, M. Skoryk, V. Yefanov, S. Schulze, D. R. T. Zahn

**Affiliations:** L. V. Pysarzhevsky Institute of Physical Chemistry, Nat. Acad. of Sci. of Ukraine 03028 Kyiv Ukraine; Semiconductor Physics, Chemnitz University of Technology 09107 Chemnitz Germany oleksandr.stroyuk@physik.tu-chemnitz.de; LLC NanoMedTech 03680 Kyiv Ukraine

## Abstract

The introduction of colloidal single-layer carbon nitride (SLCN) nanosheets at the stage of the formation of Au nanocrystals (NCs) in aqueous solutions allows the surface plasmon resonance peak position of gold/SLCN composites to be tuned in a relatively broad range of 520–610 nm. The effect is believed to originate from a strong electronic interaction between Au NCs and SLCN nanosheets attached to their surface as capping ligands and resulting in a decrease of the effective electron density on the Au NC surface. The SLCN nanosheets suppress direct interparticle interactions between Au NCs prohibiting additional plasmonic features typical for the Au NC associates. Species similar to SLCN in terms of functionalities but having no conjugated aromatic system, such as polyethyleneimine, only induce aggregation of Au NCs but do not allow the main surface plasmon resonance of the NCs to be tuned demonstrating the crucial role of electronic interaction between the NC surface and the aromatic SLCN sheets for the surface plasmon resonance tuning.

## Abstract 2

Interaction of forming Au nanocrystals with single-layer carbon nitride nanosheets allows the surface plasmon resonance peak position of gold/carbon nitride composites to be tuned in a range of 520–610 nm.

## Graphical abstract

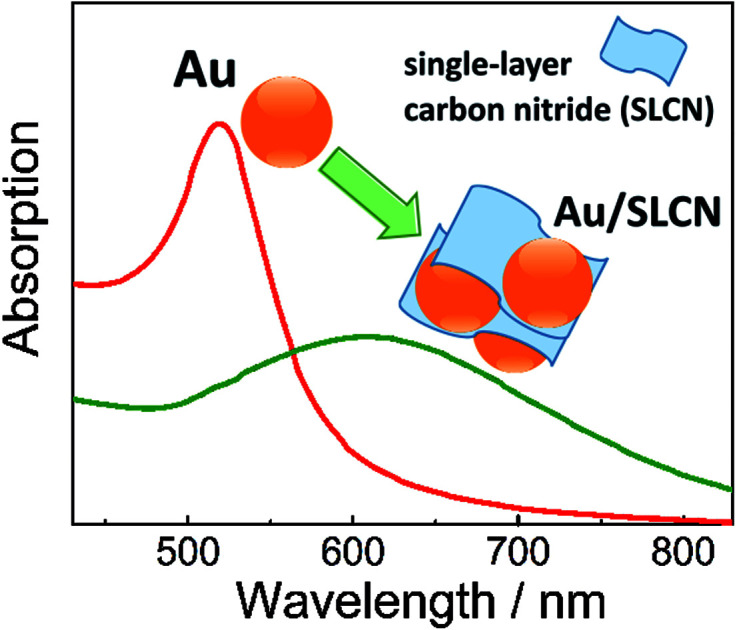

## Introduction

Gold nanocrystals (NCs) belong to the most intriguing and broadly studied objects of current research focused on nanodimensional materials.^1–5^ The vivid effect of surface plasmon resonance (SPR) in the visible spectra range typical for Au NCs defines the immense interest in their applications in various optical, opto-electronic, and sensing systems, the latter including surface-enhanced Raman spectroscopic sensing,^6,7^ optical and electrochemical sensing,^1,5,8,9^ medicine,^3–5^ and catalysis.^2^ It is natural that the issue of tuning the parameters of the SPR absorption band (SPR peak position, band shape and intensity, *etc.*) of Au NCs is critical for such applications.^10,11^

One straightforward way of tuning the SPR parameters of Au NCs, that comes from ancient times to the present day, is by changing the NC size and shape, as well as the mode of interaction between NCs. The immense research effort invested in the synthesis of Au NCs revealed promising ways of precise tailoring of both the NC size and size distribution,^1,8,10,12–15^ but showed that the position of SPR band maximum varies only modestly in a broad NC size range. For example, the variation of Au NC size by more than an order of magnitude, from 5–10 to 100–110 nm, was found to result in a “red” shift of the SPR maximum by merely ∼50–65 nm.^8,14,15^ The variation of the Au NC shape (nanorods, nanostars, nanoshells, *etc.*) was found to yield uncomparably richer plethora of SPR peaks^7,10^ ranging from 780 to 860 nm for nanorods and from 900 to 1100 nm for nanoshells.^12^ At the same time, a precise shape control is accompanied by a strong increase in the complexity of preparative procedures and sensitivity to the reproducibility as well as scalability issues.

Controlled aggregation is also an efficient way of influencing the SPR properties of Au NCs,^1,8,9,11,16–19^ however, it compromises the individual nanometer character of Au NCs, that can be crucial, for example, in bio-medical applications.^20^ In this view, the issue of tuning the SPR properties of Au NCs without altering considerably their morphology still remains a challenge.

The electronic properties of gold NCs can be strongly affected by coupling them with other conducting or semiconducting species by aligning the Fermi energies or charge transfer effects possible in such composites.^2,21–24^ Studies of Au NCs deposited on the surface of layered 2D materials, such as a-few-layer graphene, graphitic carbon nitride (GCN), layered metal dichalcogenides are of special interest because Au NCs can strongly interfere with charge transfer across the basal plane of the adjacent layers and affect the optical, catalytic, and photochemical properties of the layered host materials.^21–23^ In the particular case of GCN, which is one of the most promising 2D materials^21,25^ probed currently for catalytic and sensoric applications, the decoration with Au NCs was reported to enhance the electrochemical,^21,26^ catalytic,^21,27,28^ and photocatalytic^21,27,29–31^ activity of the layered host.

The electronic interaction between layered materials and attached Au NCs can be visualized by shifts and broadening of the SPR bands of the metal NCs.^24,26,32,33^ At that, the strongest interaction between Au NCs and layered materials is expected for single-layer species, however, the reports on such composites are still quite rare. Typically, Au NCs are coupled with graphene^34^ and graphene oxide,^24,32,33^ yielding composites with perspectives for surface-enhanced Raman spectroscopic sensors^32,33^ and catalysis.^32^ However, no reports can be found, to the best of the authors' knowledge, on composites of Au NCs with single-layer carbon nitride (SLCN) species.

Earlier we reported on the synthesis of single-layer carbon nitride *via* exfoliation of bulk GCN in hot aqueous solutions of tetraethylammonium hydroxide.^35^ The method yields stable and concentrated (up to 50 g L^−1^) aqueous colloidal solutions predominantly containing SLCN nanosheets with a thickness of 0.34–0.35 nm and a lateral size of 40–50 nm. The SLCN sheets are composed of conjugated heptazine heterocycles and reveal semiconducting properties with a bandgap of ≈2.7 eV.^35^ The incomplete character of the melamine polycondensation at the stage of the synthesis of bulk GCN as well as a partial hydrolytic decomposition of polyheptazine sheets during the exfoliation procedure result in the fact that the colloidal SLCN particles are decorated with amino-groups^35,36^ that can bind to ZnO NCs controlling their growth dynamics and final NC size.^37^ A photoluminescence study of ZnO/SLCN composites showed the unique size-dependent character of the electronic interactions occurring in such a tandem^37^ and indicated the feasibility of using SLCN as a functional ligand affecting not only the size of inorganic NCs but also their electronic properties. As a development of these studies, we report in the present paper on the influence of SLCN nanosheets on the morphology and optical characteristics of colloidal gold NCs.

## Experimental

Single-layer colloidal carbon nitride was synthesized according to our previous report^35^ by the exfoliation of bulk GCN (produced by the thermal polycondensation of melamine in air) in a boiling aqueous solution of tetraethylammonium hydroxide.

Colloidal gold was synthesized by reducing NaAuCl_4_ with ascorbic acid in alkaline aqueous solutions (pH 13). In a typical synthesis, 0.1 mL 0.1 M NaAuCl_4_ aqueous solution was added to 9.7 mL of distilled water followed by 0.1 mL 1.0 M aqueous NaOH solution and 0.1 mL 0.1 M aqueous solution of ascorbic acid. No heating procedures were applied during the synthesis. Each step of reactant addition was accompanied by intense stirring. All reagents used were supplied by Signal-Aldrich and used without any further purification.

The Au/SLCN systems were produced either *in situ* by forming Au NCs in the presence of SLCN sheets or *ex situ* by adding SLCN nanosheets after the synthesis of Au NCs. In the *in situ* method SLCN was introduced together with NaOH. For the purpose of comparison, Au NCs were also synthesized in the presence of branched polyethyleneimine (Sigma Aldrich) with a varying concentration.

Absorption spectra of colloidal solutions were recorded using a Shimadzu UV-3600 double-beam spectrophotometer in standard 2.0 mm quartz cuvettes using distilled water as a reference. Scanning electron microscopy (SEM) was performed using a Tescan Mira3 LMU microscope with an accelerating voltage of 15–20 kV in regimes of back-scattered and secondary electron registration. Transmission electron microscopy (TEM) was performed using a Philips CM 20 FEG microscope at an accelerating voltage of 200 kV. Samples for SEM and TEM images were prepared by drop-casting of Au NC colloids onto a carbon-covered silicon or copper grid, respectively, followed by drying at ambient conditions. The hydrodynamic size of colloidal Au NCs was determined by the dynamic light scattering (DLS) method using a Malvern ZetaSizer Nano at 25 °C. The samples were illuminated by a He–Ne laser with *λ* = 633 nm with the scattered light collected at 173°.

## Results and discussion

**Fig. 1:**
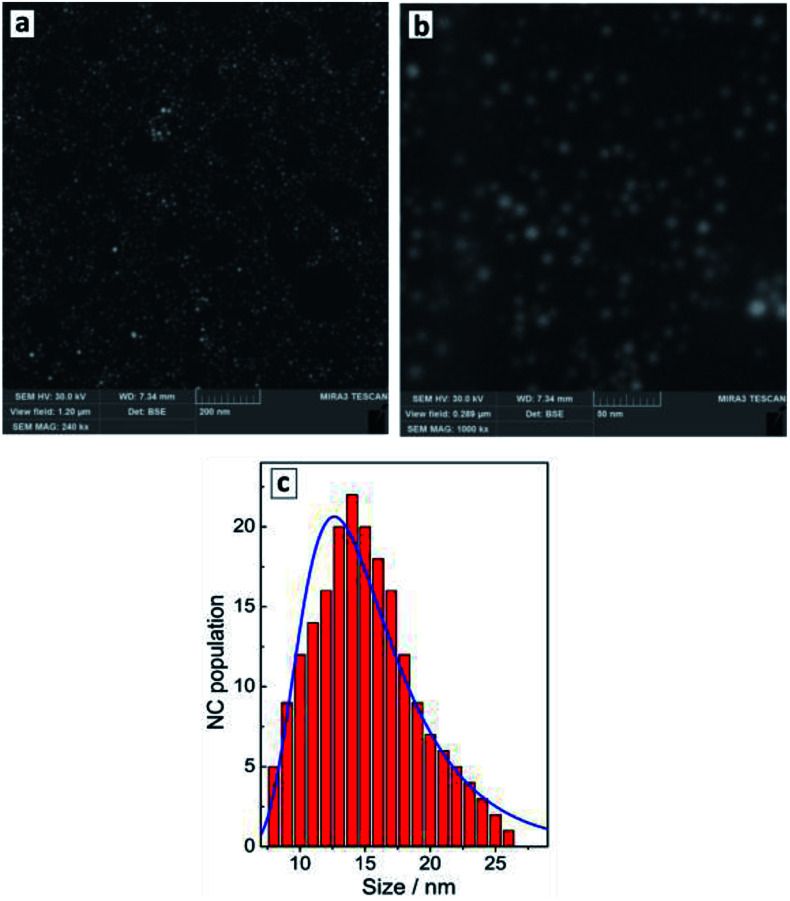
SEM images (a and b) and size distribution of Au NCs derived from SEM data (c, red bars) and from DLS measurements (c, blue line).

The reduction of tetrachloroaurate ions by ascorbic acid in alkaline aqueous solutions results in the formation of Au NCs with a shape close to spherical one and a low degree of NC aggregation (Fig. 1a). The colloidal Au NCs loose stability at decreasing pH indicating that the NCs are stabilized most probably by adsorbed hydroxide anions as well as residual ascorbate anions and oxalate anions forming as a product of the oxidation of ascorbic acid. The variety of present ligands results in a relatively broad size distribution of the final Au NCs as discussed below.

**Fig. 2:**
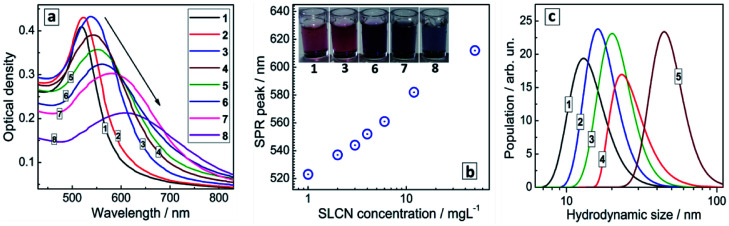
(a) Absorption spectra of Au/SLCN NCs produced *in situ* with different SLCN amounts. No SLCN (curve 1), 1 (curve 2), 2 (3), 3 (4), 4 (5), 6 (6), 12 (7), and 50 mg L^−1^ SLCN (8). The Au content is 200 mg L^−1^. (b) SPR band maximum position as a function of the SLCN content during the synthesis of Au NCs. Inset: photographs of colloidal Au NCs (1) and Au/SLCN NCs (3–8), the numbers correspond to the curve numbers in (a). (c) Hydrodynamic size distribution of Au/SLCN NCs produced at 1 (curve 1), 3 (2) 6 (3), 12 (4) 50 mg L^−1^ SLCN (5).

Colloidal Au NCs show a typical absorption spectrum (Fig. 2a, curve 1) composed of a continuous d–d transition band superimposed with a characteristic surface plasmon resonance (SPR) band peaked at 520 nm. The spectral properties of gold NC colloids do not change noticeably after a storage for many months.

The average hydrodynamic size of Au NCs determined by DLS measurements, ≈13 nm (Fig. 1c, blue curve), corresponds well to the average NC size of 14–15 nm (Fig. 1c, red bars) determined from SEM images (Fig. 1a and b). The NCs are characterized by a NC size distribution from 5–10 nm to around 25 nm showing no larger NC aggregates in the colloidal ensemble.

The introduction of SLCN nanosheets during the formation of Au NCs was found to result in a “red” shift and some broadening of the SPR band proportional to the SLCN content (Fig. 2a). As the SLCN concentration increases from 1 mg L^−1^ to 50 mg L^−1^ (0.001 M in terms of an elemental C_3_N_4_ unit of SLCN) the SPR peak position shifts from 520 nm to 610–615 nm in a logarithmic proportion to the SLCN concentration (Fig. 2b). Visually the SPR shift can be tracked by a distinct change of the color of the Au NC solution (inset in Fig. 2b). We note that all samples were produced from the same concentrated stock SLCN solution and, therefore, the morphology of SLCN nanosheets was the same for every Au/SLCN sample studied.

The average hydrodynamic size of the *in situ* produced Au/SLCN NCs shows a tendency to increase to ≈20–25 nm when varying the SLCN content from 1 to 12 mg L^−1^ (curves 1–4, Fig. 2c) and growing further to 45–50 nm at the highest SLCN concentration of 50 mg L^−1^ (curve 5, Fig. 2c). Nevertheless the colloidal Au/SLCN solutions retain a perfect aggregation stability at all SLCN contents studied. The aqueous Au/SLCN solutions can be diluted with water without losses in the colloidal stability and with the average hydrodynamic size remaining unchanged indicating that the size changes induced by SLCN addition are of a permanent character.

**Fig. 3:**
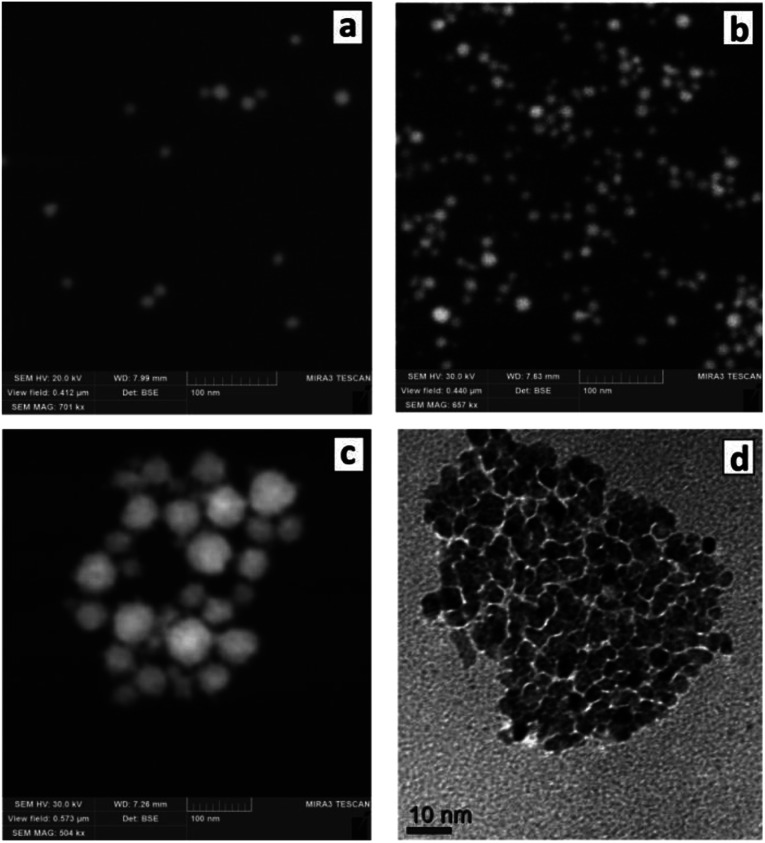
SEM (a–c) and TEM (d) images of Au/SLCN NCs produced *in situ* at 2 (a), 6 (b), and 50 mg L^−1^ SLCN (c and d). Au content is 200 mg L^−1^.

The changes of the morphology of Au NCs induced by the presence of SLCN nanosheets can also be observed by SEM that predominantly shows the individual character of Au/SLCN NCs at a low SLCN content (Fig. 3a), a tendency of Au/SLCN NCs to aggregate for intermediate SLCN concentrations (Fig. 3b), and the formation of large (50–100 nm) Au/SLCN NC aggregates at the highest SLCN content studied in the present work (Fig. 3c).

A more detailed investigation of the large aggregates by TEM (Fig. 3d) shows them to be formed by smaller Au NCs with a size of 5–7 nm (smaller than 10 nm in average) which are quite loosely aggregated indicating the presence of SLCN moieties between adjacent Au NCs preventing their tight contact.

These results reveal that the introduction of SLCN during the formation of Au NCs causes some decrease of the average size of metal NCs, the SLCN nanosheets obviously acting as an additional ligand controlling the growth of gold NCs. A similar effect of a single-layer species on the morphology of Au NCs was reported for Au/graphene composites produced by the *in situ* reduction of tetrachloroaurate anions in the presence of single and a-few-layer colloidal graphene sheets.^34^ Also, we can conclude that the increase of the hydrodynamic size of Au/SLCN NCs observed at an elevated SLCN content is caused by aggregation of separate Au NCs rather than by a real increase of the size of each Au NCs. Therefore, the “red” shift of the SPR band maximum cannot be associated with an increase of the average size of Au NCs and other reasons are obviously responsible for this effect.

The most probable reason for the “red” shift of the SPR peak of Au/SLCN NCs observed is the electronic interaction between closely bound Au NCs and SLCN sheets. The SLCN nanosheets have an aromatic character and can provide an extensive area of conjugated π-orbitals that can accept electron density from adjacent Au NCs.

Aromatic single layer moieties, such as graphene and reduced graphene oxide were reported to be perfect electron acceptors from metals and semiconductors^22–24,34,38–40^ while similar electron interaction between noble metal NCs and graphitic carbon nitride sheets were believed to be responsible for the advanced catalytic properties of such heterostructures.^21,25,30,31^ A partial shift of the electron density from the Au NCs to the SLCN sheets should result in a decrease of the surface electron density *N*_e_ and a lowering of the SPR energy. This effect can be observed as a “red” shift of the SPR absorption band (an increase of the SPR band maximum wavelength *λ*_max_), according to the well-known relationship *λ*_max_^2^ = (2π*c*)^2^*m*_e_(*ε*_0_ + 2*n*_0_^2^) × (4π*e*^2^*N*_e_)^−1^, where *ε*_0_ is a wavelength-independent component of the dielectric permeability of the metal, *c* is the light velocity in vacuum, *n*_0_ is the light refraction index.^8,12–14^

**Fig. 4:**
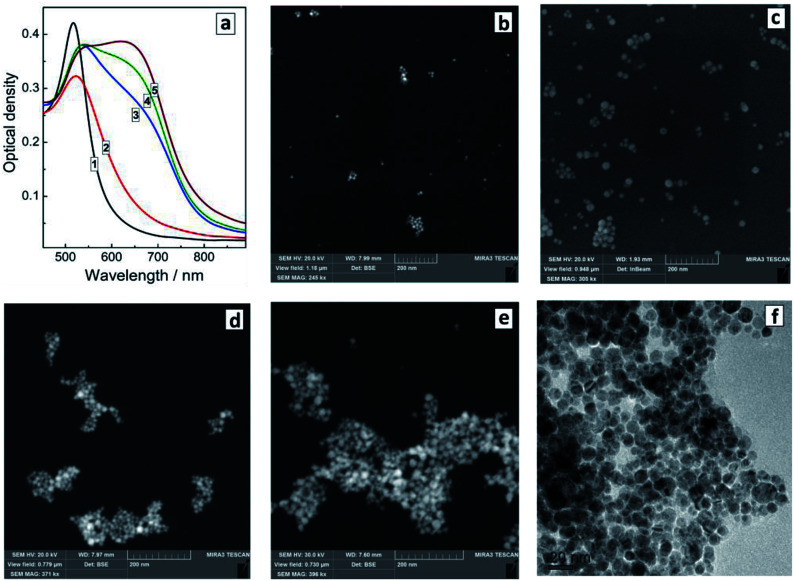
(a) Absorption spectra of Au/SLCN produced *ex situ* by adding SLCN to a Au colloid (curve 1). SLCN content is 3 (2), 6 (3), 12 (4), and 50 mg L^−1^ (5). (b–e) SEM images of *ex situ* produced Au/SLCN composites at 3 (b), 6 (c), 12 (d), and 50 mg L^−1^ SLCN (e). (f) TEM image of *ex situ* produced Au/SLCN with 50 mg L^−1^ SLCN. The Au content is always 200 mg L^−1^.

The presence of SLCN nanosheets at the moment of the formation of Au NCs is essential for the “red” SPR peak shift to be observable. We found that the optical properties of similar systems but produced by adding SLCN nanosheets to previously prepared Au NCs (*ex situ* method) are drastically different from those of the *in situ* prepared Au/SLCN composites. In particular, the addition of colloidal SLCN to pre-synthesized Au NCs results in the rise of an additional SPR peak at around 650–680 nm, while the original SPR maximum remains more or less at the same position as that of the bare Au NCs (Fig. 4a). As the content of SLCN is further elevated, the intensity of the second SPR peak grows, while the amplitude of the first SPR band remains almost unchanged. Such behavior was earlier reported for the cases of aggregation of Au NCs induced by the addition of various ligands, in particular mercapto- and amino-containing molecules.^8,9,17–19,41^ We note also that no appreciable changes in the spectral properties of Au NCs were observed upon addition of aqueous solution of only tetraethylammonium hydroxide and NaOH with no SLCN present, indicating that all above-discussed variations originate from interactions between Au NCs and SLCN nanosheets.

We showed earlier that colloidal SLCN nanosheets contain primary and secondary amino-groups arising from both the incomplete character of the melamine polycondensation and from the hydrolysis of GCN particles during the exfoliation procedure.^35,36^ We reported that the amino-groups of SLCN can bind to ZnO NCs restricting their growth and resulting in a size-selected series of ZnO/SLCN NCs.^37^ In a similar way, the amino-groups of SLCN are expected to bind to the surface of Au NCs restricting their growth in the *in situ* version of the synthesis or to form bridges between Au NCs in the *ex situ* version, resulting in the formation of Au NC aggregates. The latter expectation was proven by SEM measurements.

The introduction even of small amounts of SLCN (1–3 mg L^−1^ or 2–6 molar% of Au content) already yields small aggregates of Au NCs clearly observed along with individual Au NCs (Fig. 4b). As the SLCN content is increased, the tendency to aggregation becomes more expressed and predominantly aggregated Au NCs can be observed at 12–50 mg L^−1^ SLCN, the aggregate size increasing with an increase in the nanosheet content (Fig. 4d and e). TEM shows that aggregated Au NCs retain their original size and the association mode is quite loose (each Au NCs can be discerned on the images) indicating the presence of SLCN sheets between neighbouring Au NCs in the aggregates (Fig. 4f).

It should be noted that we did not observe any spectral signs of aggregation in the case of the *in situ* produced Au/SLCN NCs, despite the fact that the aggregation was confirmed both by DLS and SEM measurements. In particular, no distinctly shaped second SPR band can be seen in the absorption spectra of *in situ*-synthesized Au/SLCN NCs (Fig. 2a) in contrast to the *ex situ* sample (Fig. 4a). This observation can be interpreted if we assume that the SLCN nanosheets placed between neighbouring Au NCs interfere with the interparticle electromagnetic interaction and alleviate the effects of longitudinal surface plasmon resonances typically observed for Au NC aggregates.^8,9,17–19,41^ At the same time, for the case of *ex situ* produced Au/SLCN composites the contact of nanosheets with the gold NC surface is probably much weaker because the SLCN sheet need to compete with the already adsorbed ligands for the adsorption sites on the Au NC surface and, therefore, the NC surface coverage by SLCN will not be so complete as for the *in situ* samples. This fact is expected to allow for a direct electron interaction between neighbouring Au NCs and producing the second SPR absorption band in the *ex situ* Au/SLCN composites.

We believe that the damping effect of SLCN nanosheets eliminating the interparticle interactions in the *in situ* produced Au/SLCN aggregates arises from the aromatic character of the SLCN sheets that act as an electromagnetic “shield” separating the Au NCs.

To provide additional arguments for this assumption we synthesized similar composites reducing tetrachloroaurate ions in the presence of polyethyleneimine (PEI). Earlier we^42^ and other groups^41,43^ showed that PEI can act as a reductant and/or stabilizer of Au NCs also resulting in the controlled aggregation of Au NCs due to the abundant presence of amino-groups. However, in contrast to SLCN, PEI does not contain an aromatic component, the PEI molecular backbone being perfectly aliphatic. So, by comparing the influence of SLCN and PEI on the optical properties of the final *in situ* composites we can expect to discern the particular effect of aromatic subsystem on the optical spectra of Au/SLCN composite.

**Fig. 5:**
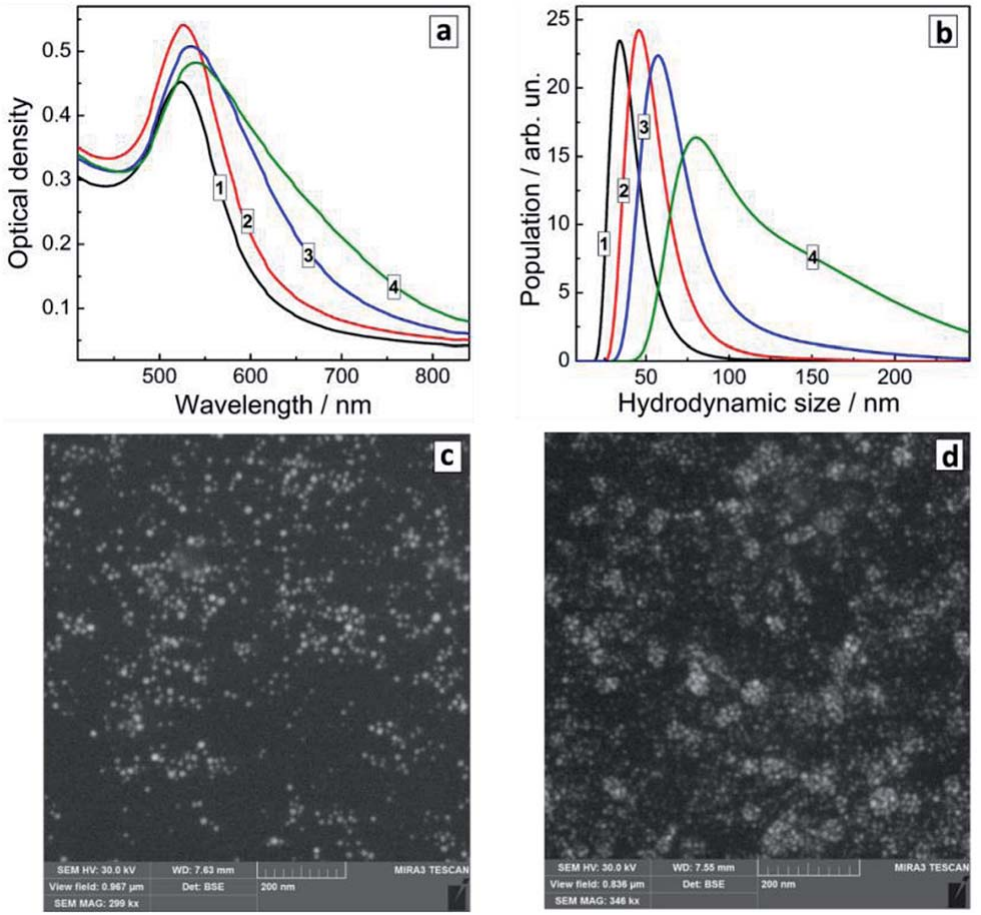
(a and b) Absorption spectra (a) and hydrodynamic size distributions (b) of colloidal Au/PEI composites produced *in situ* at 50 (curves 1), 100 (2), 150 (3), and 200 mg L^−1^ PEI (4). (c and d) SEM images of Au/PEI composites produced at 50 (c) and 200 mg L^−1^ PEI (d).

Fig. 5a shows that the effect of PEI addition during the formation of Au NCs is in general similar to the effect of the SLCN addition after the NC synthesis. In particular, we observe the gradual formation of an additional absorption shoulder as the PEI content increases, which can be interpreted as a second SPR contribution associated with the NC aggregation. At that, the main plasmon band position remains more or less stable and does not show any shifts comparable with those observed in the case of the *in situ* synthesis of Au/SLCN composites.

Dynamic light scattering measurements (Fig. 5b) confirm this assessment showing a steady tendency of Au NCs to aggregate into larger formations with the average hydrodynamic size growing to around 100 nm for the highest PEI content. The latter distribution also shows the presence of larger aggregates with a size of 150–200 nm and more (Fig. 5b, curve 4) which is in accordance with the SEM measurements (Fig. 5c and d). So, we can conclude, that PEI present at the moment of the formation of Au NCs results only in the NC aggregation by acting as a bridge bounding Au NC *via* the amino-groups and does not interact in any sense with the electronic system of Au NCs, in contrast to the SLCN nanosheets.

## Conclusions

Summarizing the above discussion, we found that the introduction of colloidal SLCN nanosheets at the stage of the formation of Au NCs allows the SPR peak position of final gold/SLCN composites to be tuned in a relatively broad range of 520–610 nm without a strong damping of the surface plasmon resonance or a decisive influence on the morphology of the Au NCs. The effect is believed to originate from a strong electronic interaction between Au NCs and SLCN nanosheets attached to their surface as capping ligands and resulting in a decrease of the effective electron density on the Au NC surface. Additionally, the SLCN nanosheets suppress direct interparticle interactions between closely positioned Au NCs prohibiting the generation of additional plasmonic features typical for the Au NC associates. Comparative experiments with SLCN nanosheets introduced after the synthesis of Au NCs showed that the presence of SLCN during the NC formation is a prerequisite for the above-discussed plasmon shift effect to be observable. Species similar to SLCN in terms of functionalities but having no conjugated aromatic system, such as PEI, can only induce aggregation of Au NCs with concomitant optical effects but do not allow the main surface plasmon resonance of the NCs to be tuned demonstrating the crucial role of electronic interaction between the NC surface and the aromatic SLCN sheets for the SPR tuning effects to be observed.

## Conflicts of interest

There are no conflicts to declare.

## Supplementary Material
